# Thoraco-laparoscopic *versus* open McKeown esophagectomy for esophageal cancer: comparative outcomes on inflammatory response and coagulation function

**DOI:** 10.1590/1414-431X2026e15544

**Published:** 2026-10-30

**Authors:** Zhengqiao Wang, Renquan Zhang

**Affiliations:** 1 Department of General Thoracic Surgery, the First Affiliated Hospital of Anhui Medical University, Hefei, Anhui Province, China

**Keywords:** Esophageal cancer, Thoraco-laparoscopic, Thoracolaparoscopy, McKeown, Inflammatory response, Coagulation function

## Abstract

We aimed to unravel the efficacy of thoraco-laparoscopic combined McKeown esophagectomy for esophageal cancer and its impact on inflammatory response and coagulation function. We retrospectively analyzed 108 esophageal cancer patients, divided into thoraco-laparoscopic (TLE) and open esophagectomy (OE) groups. Observed indicators included surgical parameters (operative time, blood loss, ICU and hospital stays), inflammatory markers [white blood cell (WBC), neutrophil percentage (NEUT%), lymphocyte percentage (LYMPH%), platelet (PLT)], coagulation profiles [prothrombin time (PT), international normalized ratio (INR), activated partial thromboplastin time (APTT), fibrinogen (FIB), D-dimer (D-D)], complications, and 1-year disease-free survival. The TLE group demonstrated reduced intraoperative blood loss, shorter ICU and total hospital stays, and lower complication rates than the OE group (P<0.05). Postoperative inflammatory and coagulation profiles also differed, with TLE showing lower WBC (11.35±2.58 *vs* 12.48±2.41 10^9^/L), NEUT% (72.28±6.62 *vs* 79.41±6.94%), PLT (233.87±19.62 *vs* 245.56±22.79 10^9^/L), PT (11.41±0.79 *vs* 12.00±1.05 s), APTT (26.70±3.60 *vs* 28.83±3.08 s), and INR (0.90±0.07 *vs* 0.95±0.10), but higher LYMPH% (21.80±4.06 *vs* 19.52±4.16 %), D-D (348.89±37.92 *vs* 315.27±36.74 μg/L), and FIB (3.95±0.56 *vs* 3.71±0.51 g/L) levels than the OE group (P<0.05). TLE was an effective and reliable approach associated with fewer complications and comparable one-year oncologic outcomes to open surgery, though its effects on coagulation parameters warrant closer clinical monitoring.

## Introduction

Esophageal cancer is a highly aggressive malignancy, ranking as the sixth leading cause of cancer-related mortality worldwide, with over 600,000 new cases reported annually (1). The disease is characterized by its poor prognosis, with a 5-year survival rate of approximately 30%, despite advancements in surgical and neoadjuvant therapies (2). Surgical resection remains the cornerstone of curative treatment for resectable esophageal cancer, with minimally invasive esophagectomy gaining widespread adoption due to its reduced postoperative complications and improved recovery times compared to open surgery (3). Among the surgical techniques, McKeown esophagectomy, involving cervical anastomosis, is commonly performed, particularly in regions where squamous cell carcinoma predominates (4). To address these issues, combined thoracoscopic and laparoscopic McKeown esophagectomy has emerged as a promising approach, offering reduced surgical trauma, less inflammatory responses, and faster recovery times (5). However, the procedure is associated with significant challenges, including a high incidence of anastomotic leakage and related complications, which can lead to severe morbidity and mortality (6). Despite the aforementioned advantages of minimally invasive techniques, the inflammatory response and coagulation dysfunction triggered by surgical trauma remain critical issues affecting patients' postoperative recovery process and prognosis (7).

Surgical trauma immediately triggers the body's stress response, in which the inflammatory response and activation of the coagulation system are intertwined, jointly influencing patients' postoperative recovery (7). Damage-associated molecular patterns released from tissue injury initiate an inflammatory cascade. If regulation is imbalanced, excessive inflammation can lead to immune dysregulation, increasing the risk of infection and multiple organ dysfunction (8-10). In addition, vascular injury exposes tissue factor, activating the coagulation pathway to control bleeding, but its excessive activation may cause microcirculatory disorders or thrombosis, further exacerbating tissue damage (8,11,12).

Therefore, identifying surgical strategies that can mitigate perioperative inflammatory responses and coagulation dysfunction is of great significance for improving the postoperative outcomes of patients with esophageal cancer. This study aimed to compare the efficacy of thoraco-laparoscopic combined and open McKeown esophagectomy for esophageal cancer, focusing on the impact of the two surgical approaches on patients' postoperative inflammatory responses and coagulation function, with the goal of providing more robust evidence-based support for optimizing surgical strategies and promoting patients' postoperative recovery.

## Material and Methods

### Ethical approval

All experimental procedures were approved by the Medical Ethics Committee of the First Affiliated Hospital of Anhui Medical University, China. Patients were notified about the information contained in the consent form. All procedures were conducted in accordance with the Declaration of Helsinki.

### Subjects

A retrospective analysis was conducted on patients with esophageal cancer admitted to the First Affiliated Hospital of Anhui Medical University from January 2021 to February 2024. A total of 108 patients met the inclusion criteria and were enrolled in this study. The patients were divided into the thoraco-laparoscopic combined McKeown esophagectomy group (TLE group) and the open McKeown esophagectomy group (OE group), with 54 cases in each group. Among them, there were 72 males and 36 females, with an average age of 57.58±10.19 years. There were 84 cases of mid-thoracic esophageal cancer and 24 cases of lower esophageal cancer. No noticeable differences were observed in the general characteristics between the TLE group and OE group (P>0.05), indicating comparability (Table 1). The flowchart of the study is shown in Figure 1.

**Figure 1:**
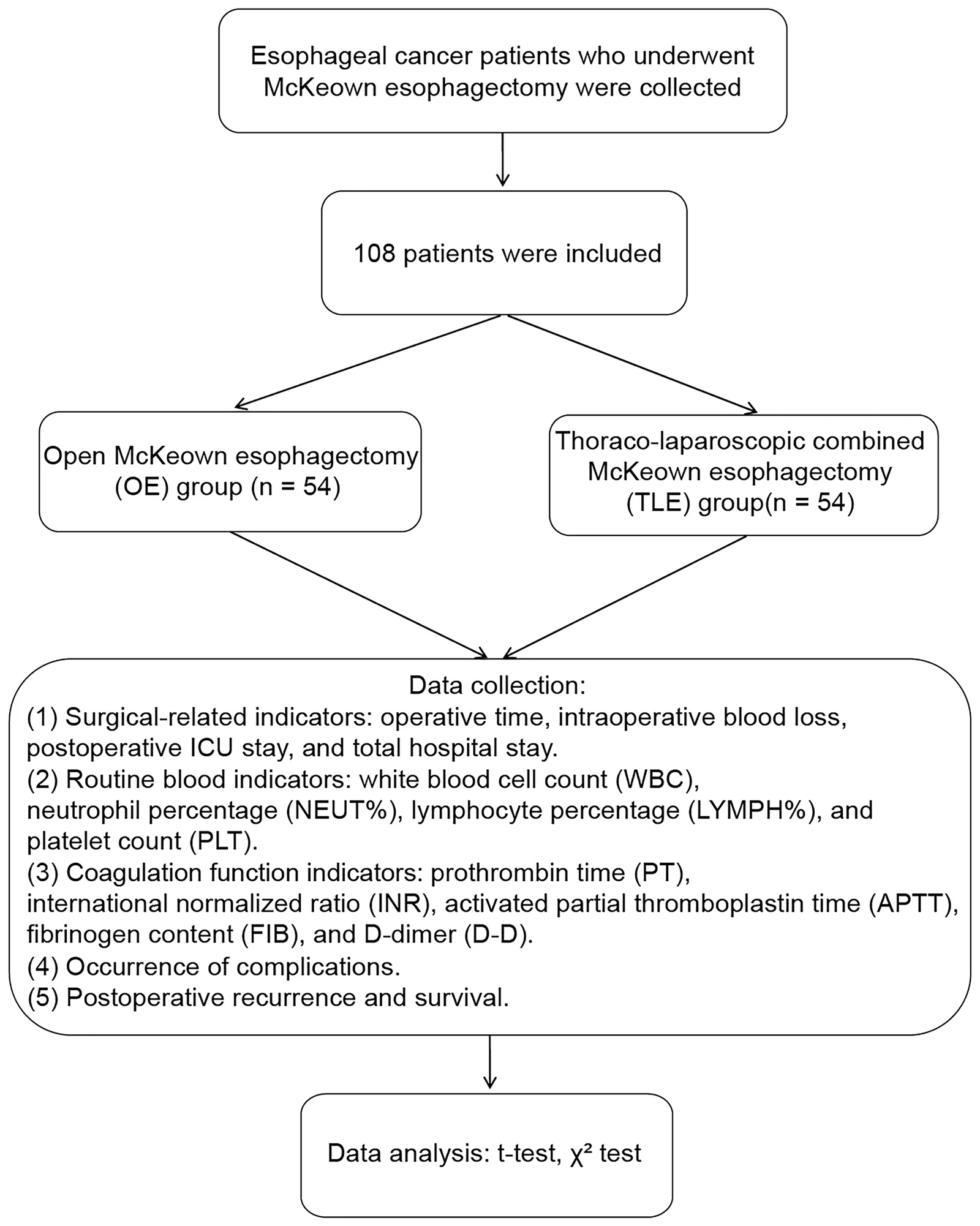
Flowchart of this study.

**Table 1:** General characteristics between the OE and TLE groups.

Indicator	OE group (n=54)	TLE group (n=54)	P
Gender			0.414
Male	34 (62.96)	38 (70.37)	
Female	20 (37.04)	16 (69.63)	
Age (years)	58.81±10.16	56.35±10.16	0.211
Body mass index (kg/m^2^)	23.36±1.75	23.23±1.86	0.714
Smoking			0.844
Yes	33 (61.11)	32 (59.26)	
No	21 (38.89)	22 (40.74)	
Alcohol consumption			0.846
Yes	31 (57.41)	30 (55.56)	
No	23 (42.59)	24 (44.44)	
Time from diagnosis to surgery (months)	10.29±7.09	10.83±6.30	0.679
Tumor location			0.643
Middle segment	43 (79.63)	41 (75.93)	
Lower segment	11 (20.37)	13 (24.07)	
Tumor diameter (cm)	3.39±0.51	3.42±0.50	0.825
ASA classification			0.414
Grade I	16 (29.63)	20 (37.04)	
Grade II	38 (70.37)	34 (62.96)	

Data are reported as n (%) or means±SD. Statistical analyses were conducted using the chi-squared test or independent samples *t*-test. OE group: open McKeown esophagectomy group; TLE group: thoraco-laparoscopic combined McKeown esophagectomy group; ASA: American Society of Anesthesiology.

Inclusion criteria: 1) Patients pathologically confirmed with esophageal cancer and clinically staged as T1-3N0-1M0 (clinical staging was determined according to the 8th edition TNM staging system of the Union for International Cancer Control/American Joint Committee on Cancer through computed tomography scans, endoscopic ultrasound, and positron emission tomography scans); 2) Patients who underwent either open thoracic McKeown esophagectomy or thoraco-laparoscopic combined McKeown esophagectomy; 3) Patients aged over 18 years; 4) Patients who did not receive preoperative radiotherapy or chemotherapy.

Exclusion criteria: 1) Patients with a previous history of thoracic or abdominal surgery; 2) Patients with postoperative recurrence; 3) Patients unable to tolerate prolonged anesthesia; 4) Patients with a history of malignancy; 5) Patients with coagulation disorders or immune system diseases; 6) Patients with cognitive disorders that prevent normal communication; 7) Patients with missing clinical data (e.g., postoperative recovery-related indicators, postoperative follow-up data, etc.).

### Surgical approach

Both surgical procedures were performed by the same team of surgeons. Preoperatively, all patients underwent routine blood tests, blood biochemistry, stool and urine routine tests, liver and kidney function tests, coagulation function tests, and immune-related examinations. All patients had a gastric tube inserted for gastrointestinal decompression and received combined intravenous general anesthesia.

The OE group underwent open McKeown esophagectomy: after general anesthesia, the patient was placed in the supine position. A main operating port was created one fingerbreadth below the umbilicus, and auxiliary operating ports were made 2.0 cm below the costal margin at the midclavicular lines bilaterally. A pneumoperitoneum was established using a needle tube (CO_2_ pressure set at 10-12 mmHg). Following the insertion of the trocar, the ultrasonic scalpel was used laparoscopically to sequentially dissect the greater and lesser curvatures of the stomach. The left gastroepiploic artery was ligated and divided using Hemlock clips. Lymph nodes along the greater and lesser curvatures, cardia, and splenic artery were dissected. The esophageal hiatus was freed, and lymph nodes in the thorax, main bronchi, and above the diaphragm were dissected. The cervical esophagus was freed, and the tumor was resected. A purse-string suture was placed, and the auxiliary port was closed. The tubular stomach was pulled along the esophageal bed to the neck, and a manual full-thickness interrupted end-to-side anastomosis was performed. After confirming no active bleeding under laparoscopic inspection, the surgical site was irrigated with saline. A nasogastric tube and feeding tube were left in place, and the anastomosis, neck, and abdominal incisions were sutured sequentially.

The TLE group underwent thoraco-laparoscopic combined McKeown esophagectomy. Under general anesthesia, the procedure was divided into the thoracoscopic and laparoscopic parts. During the thoracoscopic phase, the patient was placed in the left lateral decubitus position. A 10-mm trocar observation port was created at the 7th intercostal space of the right midaxillary line, through which the thoracoscope was inserted. A 5-mm trocar main operation port was established at the 4th intercostal space of the right anterior axillary line, and a 5-mm trocar auxiliary operation port was made at the 9th intercostal space of the right subscapular line. A pneumothorax was induced with CO_2_ pressure maintained at 6-8 mmHg. The mediastinal pleura was incised using an ultrasonic scalpel or electric hook, followed by the mobilization of the thoracic esophagus, during which care was taken to protect the vagus nerve and thoracic duct to avoid injury. Subsequently, we performed a complete lymphadenectomy of the paraesophageal, main bronchial, subcarinal, and supradiaphragmatic lymph nodes. After confirming the absence of active bleeding, the thoracic cavity was irrigated with saline, and a thoracic drainage tube was placed.

In the laparoscopic section, the patient was positioned in the supine position. A 10-mm trocar was placed below the umbilicus as the observation port, and a laparoscope was inserted. Auxiliary operation ports (5-mm trocars) were created 2.0 cm below the costal margin along the left and right midclavicular lines. A pneumoperitoneum was established with CO_2_ pressure set at 10-12 mmHg. The greater and lesser curvatures of the stomach were dissected using an ultrasonic scalpel, preserving the right gastric artery and the right gastroepiploic artery. The left gastroepiploic artery and the short gastric arteries were ligated and divided using Hemlock clips. Lymph nodes along the greater and lesser curvatures, the paracardial lymph nodes, and the lymph nodes along the splenic artery were dissected. The diaphragmatic esophageal hiatus was incised, and the abdominal esophagus was dissected to meet the thoracic section. A linear stapler was used to create a tubular stomach, preserving the blood supply from the right gastric artery. After confirming that the anastomosis was tension-free and without bleeding, the gastric tube and the feeding tube were secured. Finally, the incisions were closed sequentially, and thoracic and abdominal drainage tubes were left in place. Relevant images during thoraco-laparoscopic combined McKeown esophagectomy are shown in Figure 2.

**Figure 2:**
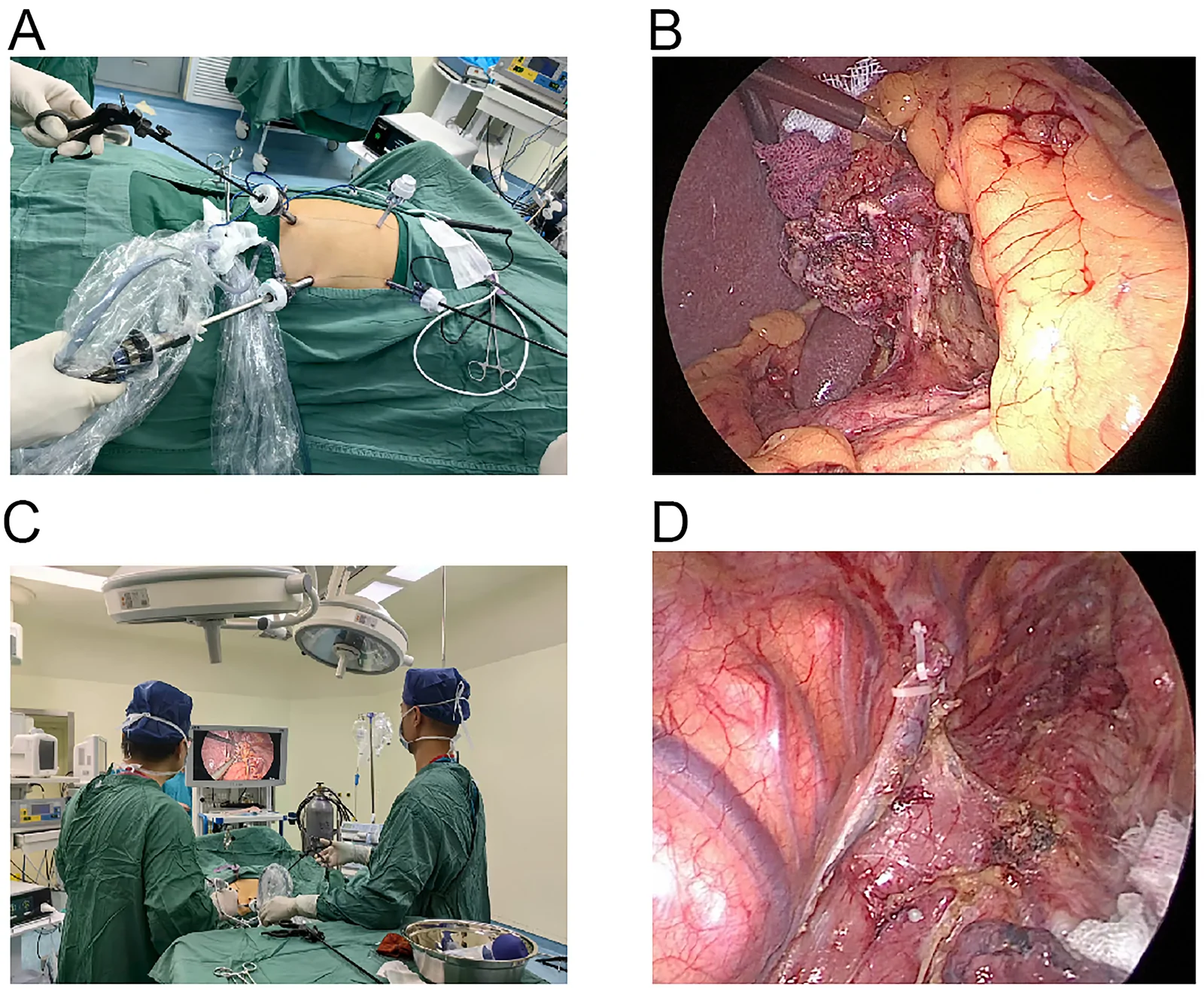
Images of thoraco-laparoscopic combined McKeown esophagectomy. **A**, Laparoscopic operating port; **B**, laparoscopic abdominal lymph node dissection; **C**, thoracoscopic operating port; **D**, thoracoscopic upper mediastinal lymph node dissection.

Both groups received routine postoperative monitoring, with close observation of vital signs, and were administered anti-infection therapy, nutritional support, and analgesic treatment. The nasogastric tube was removed 24-48 h postoperatively, and diet was gradually resumed.

### Data collection

#### Surgery-related indicators

We compared the operative time, intraoperative blood loss, postoperative ICU stay, and total hospital stay between the two groups.

#### Routine blood test indicators

White blood cell count (WBC), neutrophil percentage (NEUT%), lymphocyte percentage (LYMPH%), and platelet count (PLT) were assessed. Routine blood tests were conducted at two time points: preoperatively and postoperatively. On the day before surgery and three days after surgery, 2 mL of fasting peripheral venous blood was collected and tested using fully automated hematology analyzers (Coulter LH 750, Beckman Coulter, USA). The collection, preparation, storage, and monitoring of blood samples were carried out in strict accordance with standard operating procedures.

#### Coagulation function indicators

Prothrombin time (PT), international normalized ratio (INR), activated partial thromboplastin time (APTT), fibrinogen content (FIB), and D-dimer (D-D) were tested on the day before surgery and three days after surgery. Two mL of fasting peripheral venous blood was collected and tested using a fully automated coagulation analyzer. INR was calculated based on the PT value using the formula INR = (patient PT/12.5)ˆinternational sensitivity index (ISI)), where the ISI of the reagent was labeled as 1.2.

#### Occurrence of complications

We compared the incidence of recurrent laryngeal nerve injury (patients reported symptoms such as hoarseness, low pitch, and fatigue during prolonged speech, and laryngoscopy revealed edema and congestion of the recurrent laryngeal nerve), anastomotic leakage (full-thickness gastrointestinal defects in the esophagus, anastomosis, staples, or gastric tube, with saliva and gas observed in surgical drainage), delayed gastric emptying (characterized by epigastric fullness, nausea, vomiting with bile, gastric drainage >800 mL/day, and duration >10 days), hematoma and infection (manifested as redness, swelling, induration, subcutaneous ecchymosis, or even bloodstains at the incision site), and chylothorax (milky pleural effusion confirmed by imaging, with triglyceride level in pleural effusion ≥1.24 mmol/L) between the two groups.

#### One-year disease-free survival

All patients were followed up from the treatment date, with a postoperative follow-up period of one year, ending on February 28, 2025. During the follow-up period, the recurrence, metastasis, or death from any cause in patients were recorded every three months through outpatient reviews, home visits, and telephone follow-ups.

### Statistical methods

Since this study had a retrospective design, the analysis was limited to the number of eligible patients during the study period, and thus no sample size calculation was performed. Data processing was performed using SPSS 26.0 statistical software (IBM, USA). Measurement data are reported as means±SD, with intergroup comparisons conducted using an independent samples *t*-test and intragroup comparisons using a paired *t*-test. Enumeration data are presented as frequency/rate (%) and were tested by the chi-squared test or the continuity-adjusted chi-squared test. One-year disease-free survival was calculated by Kaplan-Meier (K-M) survival analysis, and the significance was evaluated by the log-rank test. A P-value <0.05 was considered statistically significant.

## Results

### Surgery-related indicators

There was no statistically significant difference in the operative duration between the TLE group and the OE group (P>0.05). However, compared with the OE group, the TLE group demonstrated reduced postoperative ICU stay, total hospitalization time, and intraoperative blood loss (P<0.05) (Table 2).

**Table 2:** Analysis of surgery-related indicators between the OE and TLE groups.

Indicator	OE group (n=54)	TLE group (n=54)	P
Operative time (min)			
Chest	117.61±12.52	119.09±13.19	0.551
Abdomen	120.43±11.33	123.04±12.11	0.250
Intraoperative blood loss (mL)			
Chest	109.91±10.36	104.63±7.20	0.003
Abdomen	128.89±10.45	104.93±7.21	<0.001
Postoperative ICU stay (d)	4.85±0.92	4.44±0.96	0.027
Total hospital stay (d)	11.26±1.26	10.43±0.96	<0.001

Data are reported as means±SD. Statistical analyses were performed using the independent samples *t*-test. OE group: open McKeown esophagectomy group; TLE group: thoraco-laparoscopic combined McKeown esophagectomy group; ICU: intensive care unit; d: day.

### Routine blood test indicators

Preoperatively, no notable differences were observed in WBC, NEUT%, LYMPH%, or PLT between the two groups (P>0.05). Postoperatively, WBC levels increased in both groups compared to preoperative levels, while the NEUT%, LYMPH%, and PLT in both groups decreased compared to preoperative levels (P<0.05); the WBC, NEUT%, and PLT levels in the TLE group were lower than those in the OE group, while the LYMPH% level was higher than that in the OE group (P<0.05) (Table 3).

**Table 3:** Comparison of routine blood test indicators between the OE and TLE groups.

Indicator	OE group (n=54)	TLE group (n=54)	P
WBC (10^9^/L)			
Preoperative	7.12±2.07	7.18±2.15	0.883
Postoperative	12.48±2.41*	11.35±2.58*	0.020
NEUT% (%)			
Preoperative	61.76±7.82	62.59±6.71	0.555
Postoperative	79.41±6.94*	72.28±6.62*	<0.001
LYMPH% (%)			
Preoperative	28.70±7.88	28.20±7.49	0.736
Postoperative	19.52±4.16*	21.80±4.06*	0.005
PLT (10^9^/L)			
Preoperative	258.43±24.15	258.91±23.76	0.917
Postoperative	245.56±22.79*	233.87±19.62*	0.005

Data are reported as means±SD. Statistical analyses were conducted using either an independent samples *t*-test (for unpaired data) or a paired *t*-test (for paired data). *P<0.05 compared with preoperative values. Normal reference range: WBC 4.0-10.0×10^9^/L; NEUT% 0.3-0.7; LYMPH% (%) 0.20-0.40; PLT 100-300×10^9^/L. OE group: open McKeown esophagectomy group; TLE group: thoraco-laparoscopic combined McKeown esophagectomy group; WBC: white blood cell count; NEUT%: neutrophil percentage; LYMPH%: lymphocyte percentage; PLT: platelet count.

### Coagulation function indicators

Preoperatively, there were no differences in PT, INR, APTT, FIB, and D-D between the two groups (P>0.05). Postoperatively, PT and APTT decreased in both groups compared to preoperative values, with the TLE group showing lower values than the OE group (P<0.05). D-D, INR, and FIB increased in both groups relative to preoperative values, with the TLE group exhibiting higher values than the OE group (P<0.05) (Table 4).

**Table 4:** Comparison of coagulation function indicators between the OE and TLE groups.

Indicator	OE group (n=54)	TLE group (n=54)	P
PT (s)			
Preoperative	12.61±0.88	12.76±0.87	0.380
Postoperative	12.00±1.05*	11.41±0.79*	0.001
INR			
Preoperative	1.01±0.09	1.03±0.08	0.373
Postoperative	0.95±0.10*	0.90±0.07*	0.001
APTT (s)			
Preoperative	30.19±3.24	30.07±3.35	0.861
Postoperative	28.83±3.08*	26.70±3.60*	0.001
FIB (g/L)			
Preoperative	2.34±0.28	2.30±0.22	0.336
Postoperative	3.71±0.51*	3.95±0.56*	0.020
D-D (μg/L)			
Preoperative	144.14±30.02	145.19±28.88	0.854
Postoperative	315.27±36.74*	348.89±37.92*	<0.001

Data are reported as means±SD. Statistical analyses were conducted using either an independent samples *t*-test (for unpaired data) or a paired *t*-test (for paired data). *P<0.05 compared with preoperative values. Normal reference ranges: PT: 11-14 s; INR: 0.8-1.2; APTT: 20-40 s; FIB: 2-4 g/L; D-Dimer (D-D): <0.5 mg/L. OE group: open McKeown esophagectomy group; TLE group: thoraco-laparoscopic combined McKeown esophagectomy group; PT: prothrombin time; INR: international normalized ratio; APTT: activated partial thromboplastin time; FIB: fibrinogen content; D-D: D-dimer.

### Occurrence of complications

Postoperatively, the complication rate in the TLE group was lower than that in the OE group (P<0.05) (Table 5).

**Table 5:** Comparison of complications between the OE and TLE groups.

Group	Recurrent laryngeal nerve injury	Anastomotic leakage	Delayed gastric emptying	Hematoma and infection	Chylothorax	Total incidence rate
OE group (n=54)	1 (1.85)	2 (3.70)	3 (5.56)	3 (5.56)	1 (1.85)	10 (18.52)
TLE group (n=54)	0 (0.00)	0 (0.00)	1 (1.85)	1 (1.85)	0 (0.00)	2 (3.70)
P	-	-	-	-	-	0.032

Data are reported as n (%). Statistical analyses were conducted using the continuity-adjusted chi-squared test. OE group: open McKeown esophagectomy group; TLE group: thoraco-laparoscopic combined McKeown esophagectomy group.

### One-year disease-free survival

In the OE group, one case of left supraclavicular lymph node metastasis (LNM) occurred at 6 months postoperatively, and one case of abdominal LNM occurred at 11 months postoperatively, with no fatal cases. In the TLE group, one case of left cervical LNM occurred at 8 months postoperatively, with no fatal cases. The differences in 1-year disease-free survival between the OE group and TLE group were not significant (log-rank P=0.560) (Figure 3).

**Figure 3:**
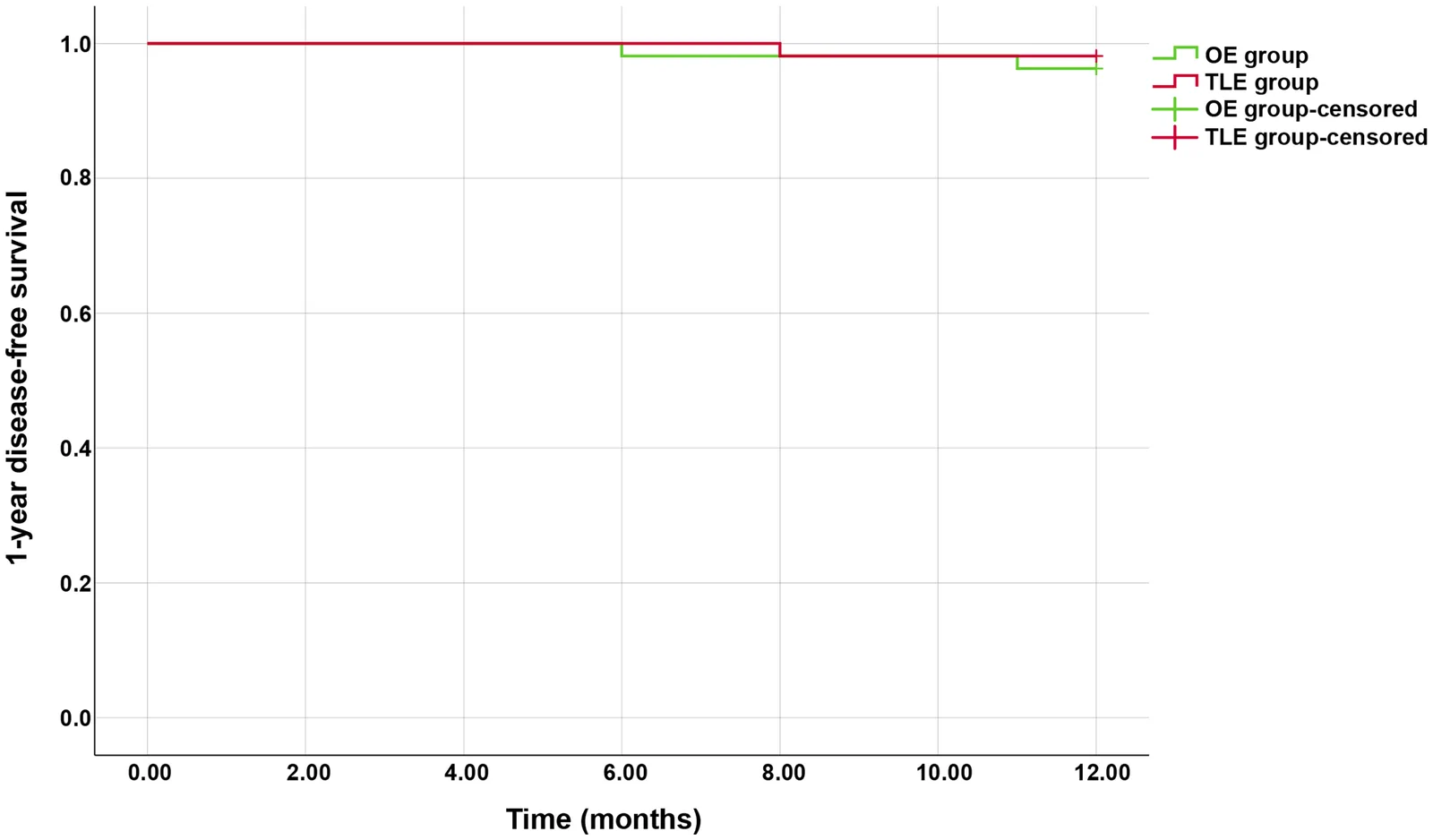
Kaplan-Meier plot of 1-year disease-free survival rates in the two groups of patients. OE: open esophagectomy; TLE: thoraco-laparoscopic combined McKeown esophagectomy.

## Discussion

Esophageal cancer remains a significant global health challenge, ranking as the sixth leading cause of cancer-related mortality worldwide (13). The development of minimally invasive techniques, such as combined thoracoscopic and laparoscopic McKeown esophagectomy, aims to reduce surgical trauma and improve postoperative outcomes (5). The present study demonstrated several findings regarding combined thoracoscopic and laparoscopic McKeown esophagectomy for esophageal cancer.

Compared with the OE group undergoing open McKeown esophagectomy, the TLE group, which underwent combined thoracoscopic and laparoscopic McKeown esophagectomy, demonstrated reduced postoperative ICU stay, total hospitalization time, and intraoperative blood loss. This result suggests that the surgical approach in the TLE group had advantages in reducing surgical trauma and promoting postoperative recovery in patients. These results align with previous studies that have shown minimally invasive McKeown esophagectomy to be associated with better surgical outcomes, including reduced morbidity and shorter recovery times (14). Our results are in accordance with one study that reported that minimally invasive esophagectomy in elderly patients was associated with lower overall morbidity and shorter hospital stays (15).

Regarding inflammatory response, we observed that in the postoperative TLE group, WBC, NEUT%, and PLT were all lower than those in the OE group, while LYMPH% was higher than that in the OE group. This indicated that this surgical approach may cause relatively less inflammatory stimulation to the body. This study also found that both groups showed decreased PT and APTT postoperatively, but the TLE group had lower values. This aligns with findings from Chen et al. (16) who reported notable alterations in coagulation parameters in esophageal cancer patients. This indicates that blood coagulability of patients in the TLE group is higher than that in the OE group. Notably, the shortening of PT and APTT was more pronounced in the TLE group, a phenomenon with significant clinical implications. PT and APTT are indicators reflecting the overall function of the exogenous and endogenous coagulation pathways (17,18). Their shortening typically suggests that the blood is in a hyper-reactive state, i.e., a hypercoagulable state. The changes in the TLE group in this study imply that although minimally invasive surgery reduces surgical incision trauma, physiological disturbances caused by factors such as the establishment of pneumoperitoneum, special positioning, and prolonged delicate operations may still trigger stronger coagulation activation than expected (in this study, no deep vein thrombosis was observed in either group). A previous study has confirmed that carbon dioxide pneumoperitoneum during laparoscopic surgery can lead to significant changes in coagulation parameters, potentially inducing a hypercoagulable state (19). This early postoperative hypercoagulable state is an independent risk factor for venous thromboembolism (VTE), including deep vein thrombosis and pulmonary embolism, in surgical patients (20). Therefore, the results of this study remind clinicians to maintain vigilance regarding VTE prevention after TLE, despite the minimally invasive nature of the surgery. When formulating anticoagulation strategies, clinicians should comprehensively consider patients' coagulation monitoring indicators, bleeding risk, and postoperative recovery status to determine the optimal timing and dosage of anticoagulant drug initiation, achieving a balance between preventing thrombosis and controlling the risk of anastomotic bleeding. Future large-scale prospective studies are needed to explore the clinical value of risk stratification and intervention for this postoperative hypercoagulable state.

Additionally, the lower incidence of postoperative complications in the TLE group supports the growing body of evidence that minimally invasive approaches reduce morbidity. That is, minimally invasive McKeown esophagectomy is associated with better surgical outcomes, including lower incidence of complications (14). Additionally, it has been reported that minimally invasive McKeown esophagectomy can decrease respiratory complications (21). Regarding long-term outcomes, we found no significant difference in 1-year disease-free survival between the two groups, highlighting that the minimally invasive approach does not compromise oncological outcomes. This aligns with previous findings that minimally invasive esophagectomy reduces respiratory morbidity without compromising oncological outcomes (21).

However, this study has the following limitations. First, as a retrospective, single-center study, its design may lead to potential selection bias, affecting the robustness of the results. Second, due to the retrospective design of the study, only eligible patients during the study period were included in the analysis, so no a priori sample size calculation was performed. Third, the follow-up period of this study was relatively short, and the assessment of long-term survival rates and long-term complications was inadequate.

In conclusion, compared with open surgery, thoraco-laparoscopic combined McKeown esophagectomy for esophageal cancer can shorten hospital stays, reduce intraoperative bleeding, lower the incidence of complications, and alleviate postoperative inflammatory responses; however, the one-year oncological prognosis is comparable. Notably, this study also observed an impact of minimally invasive surgery on postoperative coagulation function, suggesting the need to strengthen perioperative monitoring and management of coagulation status in clinical applications. Future multi-center prospective studies with longer follow-up periods and larger sample sizes are needed to further validate these findings and optimize perioperative management strategies.

## Data Availability

All data generated or analyzed during this study are included in this published article.
